# Design, Preparation and Characterization of Nationally Representative Synthetic Food Waste for Reproducible Waste Valorization Research

**DOI:** 10.3390/mps9030093

**Published:** 2026-06-10

**Authors:** Ryan Scott Anderson, Sybil Sharvelle, Susan K. De Long

**Affiliations:** Department of Civil and Environmental Engineering, Colorado State University, Fort Collins, CO 80523, USA; ryan.scott.anderson@colostate.edu (R.S.A.); sybil.sharvelle@colostate.edu (S.S.)

**Keywords:** food waste, digestion, valorization, circular economy, resource recovery

## Abstract

Food waste is a readily digestible and fermentable feedstock for waste to energy bioprocesses. Approximately one third of food is wasted, thus making improvements in food waste valorization is essential for a circular economy. Laboratory results must be reproducible and as representative of scaled performance as possible to facilitate knowledge sharing between research groups. Food waste used in laboratory studies is often collected in situ or overly simplistic synthetic mixtures are used. Food waste collected in situ from any one local source at a single time point (e.g., grab samples from a cafeteria or restaurant) are not reproducible or nationally representative; additionally, overly simple synthetic mixtures are reproducible, but lack the complexity of real food waste and are not nationally representative. Thus, an adequately complex, reproducible, and nationally representative food waste recipe is needed to standardize the feedstocks used in laboratory scale food waste digestion and fermentation studies. In this work, we developed a food waste recipe made from widely and commercially available ingredients which is based on national-scale food wastage data in the United States. The nationally representative food waste mixture was 45.4% carbohydrates, 32.5% lipids, and 13.4% proteins. The biomethane potential was 495 ± 44 mL CH_4_/g VS and the food waste mixture was suitable for use in low-pH bench-scale arrested anaerobic digesters. This design approach can be adapted for other regions and countries where food loss data are available.

## 1. Introduction

The valorization of food waste via conversion into energy and chemicals is a key research area that moves our world closer to a sustainable circular economy [1]. In the United States, food waste represents 21.6% of municipal solid waste (63.2 tons annually), of which 55.9% is landfilled [2]. Food waste results in economic and resource losses [3], and current disposal methods contribute to pollution and greenhouse gas emissions [3,4,5]. While food waste should be reduced, waste will remain in a modern food supply chain, which prioritizes food security and aesthetics over efficiency.

Anaerobic digestion (AD) and arrested anaerobic digestion (AAD) are two waste valorization processes for the conversion of food waste to energy and value-added products. AD is an established technology which converts organic substrates into energy in the form of methane via methanogenesis. AAD, an emerging technology, arrests methanogenesis during digestion to produce carboxylic acids instead. The carboxylic acids can be used to produce biofuels, lubricants, and other petroleum replacement products [6]. Both technologies require economic improvements to promote widespread adoption at scale for food waste valorization.

Technology advances require laboratory-scale studies, and expanded comparison of results across research groups would facilitate progress. Food waste characteristics, such as volatile solids, chemical oxygen demand (COD), carbon to nitrogen ratio (C:N), content of carbohydrates, and lipids and proteins affect the performance of AD [7,8,9] and AAD systems [10]. To enable meaningful comparison across studies, feedstock composition must be consistent to minimize confounding variability. Standardizing feedstock within and across laboratories will improve reproducibility in laboratory-scale food waste valorization research. This underscores the need for a representative and reproducible synthetic food waste substrate.

To date, food wastes used in laboratory studies are either collected from real world sources, such as university dining halls or hotels [11,12], or are synthetic, recipe-based food waste mixtures [13,14,15]. Both real-world sources from a single location and simple synthetic mixtures lack the representativeness of food waste composition at regional or national scales. The composition of real-world food wastes collected at a small scale (e.g., for laboratory-scale experiments) varies by locale and time [12], which limits reproducibility. Simple synthetic food waste mixtures (e.g., containing only four foods) are reproducible [14], but these recipes do not capture the complex composition of real food waste and are not representative of national food loss data.

The goal of this method development was to design, produce, and characterize a synthetic food waste that is reproducible and nationally representative for the United States. We propose that synthetic food waste recipes should be standardized to improve reproducibility across studies. Given substantial differences in food waste composition across countries, such standardization is most appropriately developed at national or regional scales [16]; accordingly, this work focuses on a formulation representative of the United States. We propose that this data-driven synthetic food waste production method be used for laboratory food waste digestion studies to facilitate a cross comparison of results, and support research advances in waste valorization bioprocesses.

## 2. Materials and Methods

### 2.1. Design of a Nationally Representative Synthetic Food Waste Mixture

To develop a nationally representative and reproducible mixture, national food loss data were used to estimate the average composition of food waste in the United States. Food waste has been defined as food losses occurring at the retail and consumer levels [17,18], and food loss data for these categories were used to determine the percentage of total food waste in the United States by food category. National food loss data were obtained from the U.S. Department of Agriculture’s Economic Research Service Loss-Adjusted Food Availability (LAFA) Data Series (www.ers.usda.gov/data-products/food-availability-per-capita-data-system; accessed 16 March 2023) [19]. Foods were grouped into nine major categories: grains; fruits; vegetables; dairy products; meat; eggs; tree nuts and peanuts; added sugars and sweeteners; and added fats and oils. To calculate the percentage contributions to the total mass, retail and consumer level losses were summed by food category and divided by the total loss of the sum of retail and consumer levels. Representative foods were then chosen for each category and were selected to be relatively low cost and readily available. Each category comprised different food types, and for some categories (e.g., vegetables) no specific food dominated. Thus, chosen foods were not necessarily the most abundant wasted food in each category. A total of twenty-one foods were selected across the nine major LAFA food groups. The resultant synthetic food waste mixture composition is shown in Table 1.

This approach provides a data-driven foundation for the composition of a nationally representative synthetic food waste. In contrast, prior studies have offered limited justification for the formulation of synthetic food waste recipes, which relied on only a few ingredients and therefore lacked compositional complexity [13,14,15]. Establishing a standardized recipe that can be readily adopted by other researchers reduces preparation time for future studies and enhances experimental reproducibility across laboratories.

### 2.2. Preparation of Synthetic Food Waste Mixture

**Figure A1:**
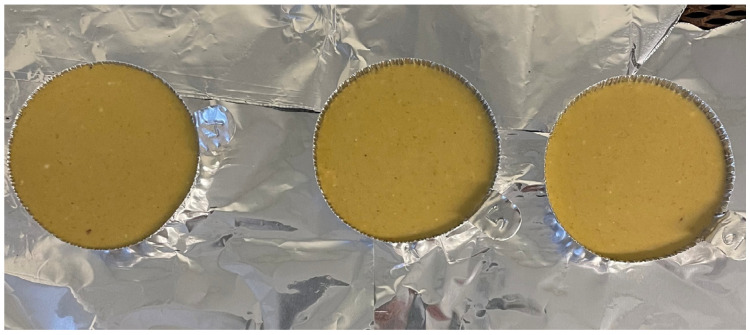
Food waste mixture in aluminum weighting dishes after blending ingredients in batches and mixing batches together. A homogeneous mixture was achieved using this approach.

To calculate a food mass for each category, a total mass for a food waste mixture batch was determined based on the mass of eggs. The mass of two eggs, shells included, was measured and multiplied by their relative fraction to calculate total mass. Inedible portions, such as peels and eggshells, were included as these components are discarded with food waste. The masses of other foods were calculated by multiplying the total mass by the percentage of total loss for the food group. Where multiple foods were selected to represent a group, the percentage of total loss was divided evenly across each food, i.e., the 13% grain products were split to be 6.5% each for oatmeal and cereal. Canned fruits and vegetables were not strained, and a proportional amount of liquid was added with the solids to be representative of the can’s contents. All foods were used fresh as purchased (i.e., grains and raw meats were uncooked while canned vegetables had been cooked). Foods were roughly chopped, where applicable, using a knife and cutting board prior to blending in batches using a VitaMix E310 (Vitamix, Cleveland, OH, USA) on high setting for 60 s per batch. Four liters of deionized water were used in conjunction with the foods to ensure smooth blending and prepare a homogenous mixture. Multiple blended batches were combined and mixed in a five-gallon bucket before aliquoting into one-gallon freezer bags for storage at −20 °C prior to further use and analysis. The food waste mixture in aluminum weighing dishes is shown in the Appendix A (Figure A1).

### 2.3. Synthetic Food Waste Validation Methods

#### 2.3.1. Calculation of Theoretical Biochemical Composition and Chemical Oxygen Demand

The USDA FoodData Central database (https://fdc.nal.usda.gov; accessed 9 December 2025) was used to calculate theoretical values for the biochemical composition and chemical oxygen demand (COD) of the food waste mixture. The average carbohydrate, protein, and fat (lipid) content of each food was obtained from the database and used to determine grams added for each food based on per serving information. From food composition data, theoretical COD was calculated using the online calculator developed by Davis et al. [20] (https://github.com/tldavi25/Food-COD-and-Eg-Calculator; accessed 9 December 2025).

#### 2.3.2. Chemical and Macromolecule Composition Analyses

Total and volatile solids were measured following APHA standard method 2540G [21] using five technical replicates. Bulk density was measured using a one-liter synthetic food waste sample and three technical replicates. Total and soluble COD were measured by the colorimetric method using Hach TNT 824 (Hach, Loveland, CO, USA) and a Hach DR3900 spectrophotometer. Prior to analysis, one gram of food waste mixture was diluted to a total volume of 25 mL with deionized water and shaken vigorously, ~20 s. Soluble COD (sCOD) was measured using the same protocol, but the diluted food waste was filtered through a 0.22 µm filter prior to analysis.

A modified version of previously published analytical procedures for biomass compositional analysis was followed [22] and was performed on duplicate food waste samples.

Modifications included drying the food waste mixture at 60 °C for 72 h prior to analysis, and the addition of a hexane extraction to measure lipids prior to water and ethanol extractions. Materials which were soluble in hexane were characterized as lipids. To determine protein content, a nitrogen-to-protein conversion factor of 4.6 was used.

Samples for carbon and nitrogen analysis were dried at 105 °C for 48 h prior to analysis. Samples were then analyzed for carbon and nitrogen using a LECO CHN 628 series (LECO Corporation; St. Joseph, MI, USA) using a LECO approved method titled “Determination of Carbon, Hydrogen, and Nitrogen in Biomass”. A sample size of 30–40 mg was wrapped into a tin cup prior to CHN analysis. The CHN analysis parameters are as follows: combustion temperature of 950 °C and an afterburner temperature of 850 °C. The burn profile is as follows: step 1: high furnace flow for 40 s; step 2: medium furnace flow for 30 s; and step 3: high furnace flow for 30 s. The ballast has an equilibration time of 30 s with a 300 s not filled timeout. The aliquot loop has a fill pressure drop of 200 mmHg with an equilibration time of 8 s. This method is calibrated with EDTA (LECO Corporation; St. Joseph, MI, USA).

#### 2.3.3. Biochemical Methane Potential

The biochemical methane potential (BMP) assay was used to characterize the food waste mixture following previously published methods [23]. Triplicate 500 mL bottles, with 1-L gas-tight Tedlar foil bags (Restek, Bellefonte, PA, USA ) for gas collection, were incubated on a shaker table set to 150 rpm in a 35 °C temperature-controlled room. Bottles and bags were leak tested prior to BMP tests. Mesophilic AD sludge from the Drake Water Reclamation Facility (Drake WRF) in Fort Collins, CO, USA was used as an inoculum source. Inoculum-only negative controls were used to determine the amount of methane produced by the inoculum alone. Positive controls using micronized cellulose (Sigma Aldrich, Burlington, MA, USA, MKCW3728) were used to confirm a viable inoculum source. For the food waste BMP assays, an inoculum to substrate ratio of 1.2 VS:VS was used, corresponding to 22.2 g food waste, 375 mL AD sludge inoculum, and 125 mL trace metals solution prepared as per [24]. Gas volume in the gas bags was measured every other day using a syringe. The methane content of the gas was measured using a gas chromatograph (HP Agilent 6890 G1530A; Agilent Technologies, Santa Clara, CA, USA) equipped with a Restek Q-bond 1074847 column and a thermal conductivity detector. Helium was used as a carrier gas at a flow rate of 133.7 mL/min and an oven temperature of 50 °C.

The BMP assays were terminated when daily methane production was <1% of the total cumulative production for three consecutive days. This occurred on day 23. To calculate methane yield (mL CH_4_/g VS), all gas volume measurements were converted to standard temperature and pressure and multiplied by the methane content. Methane produced by negative controls was averaged across triplicates and subtracted from experimental reactors and positive controls to account for methane originating from the inoculum. Yields were then calculated by dividing by feedstock mass.

#### 2.3.4. Batch Arrested Anaerobic Digestion

The performance of the food waste mixture in AAD was evaluated by measuring carboxylic acid titers, profiles, and yields. Three groups were operated in triplicate: food waste mixture inoculated with AD sludge from Drake WRF, food waste mixture alone, and AD sludge inoculum control. The experiment duration was fifteen days. Reactors in the food waste only and food waste + sludge groups were fed 25 mL of food waste mixture at days 0, 5, and 10. Table 2 outlines the reactor set up.

AAD reactors were set up in glass GL80 bottles (DWK Life Sciences, Wertheim, Germany) with in-situ pH probes (Atlas Scientific, Long Island City, NY, USA) and housed in a 35 °C constant temperature room. Reactor pH was maintained at 5.0 using an automated dosing system delivering 1.0 N sodium hydroxide and 1.0 N hydrochloric acid. Every ten minutes acid or base was added if the pH changed more than 0.2 units from the set point of 5.0. Carboxylic acid titers were measured in the sludge and food waste mixture at day 0 and from the bioreactors at day 15. Prior to carboxylic acid measurement, samples were centrifuged at 10,000× *g* for 10 min and filtered through 0.22 µm syringe filters. Carboxylic acids were measured by high-performance liquid chromatography using an Agilent 1200 series and an Aminex HPX-87H column (Bio-Rad, Hercules, CA, USA). The mobile phase was 0.01 M H_2_SO_4_. Instrument parameters were 6.0 µL injection, 0.6 mL/min 0.01 M H_2_SO_4_ at 80 °C isocratic, RID detection at 55 °C, and a needle wash position of 100. The acids identified were fatty acids of chain lengths C2–C7, succinic acid, and lactic acid; ethanol was additionally measured.

## 3. Results and Discussion

The synthetic food waste mixture was 25.0 ± 0.1% total solids and 24.2 ± 0.1% volatile solids with a density of 1040.00 ± 8.51 g/L (averages ± standard deviations of quintuplicates). The mixture reported herein was blended to form a homogenous mixture for use in batch reactors and subsequent characterizations. However, ingredients can be left whole or roughly chopped to be representative of food waste as collected. Use of the LAFA database to estimate the composition of food waste has previously been reported [25]; however, prior work focused on plate waste only and thus was not directly applicable to waste valorization research. Here we build on prior work to develop a synthetic food waste representative of collection at municipal scale.

### 3.1. Theoretical and Measured Chemical Composition

**Figure 1:**
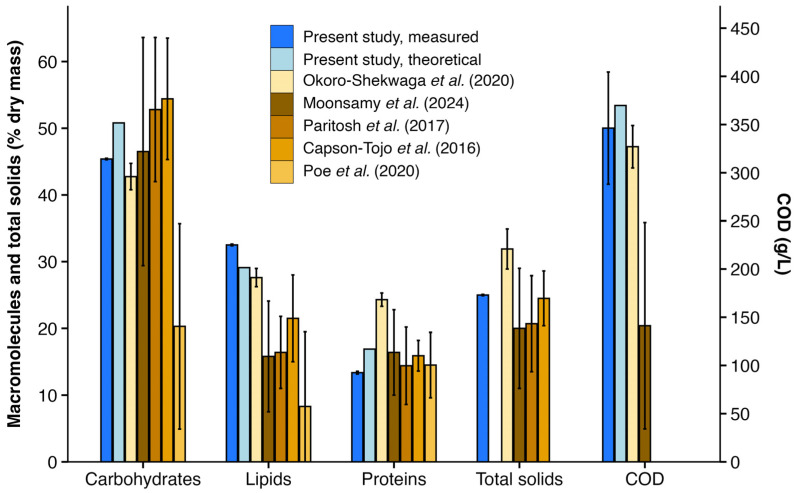
Measured and theoretical composition of the representative food waste mixture compared to literature values. Measured values presented are the average of duplicate measurements for macromolecules and triplicate measurements for COD. Error bars represent the range of duplicates for macromolecules and the standard deviation for COD. Error bars of literature values ([12,16,30,31,32]) are one standard deviation from the mean.

**Figure 2:**
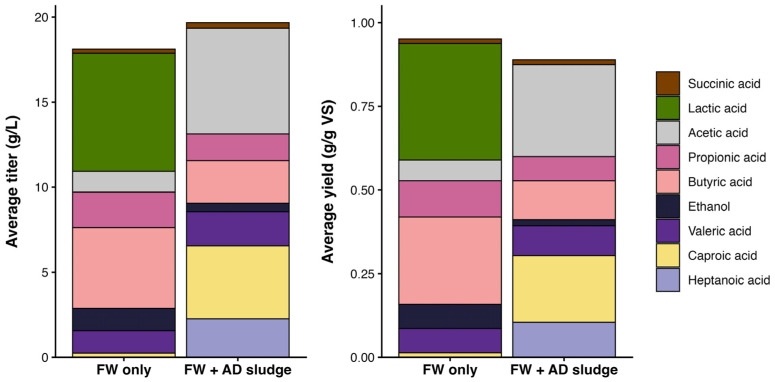
Average carboxylic acid titers and yields of triplicate reactors at the final timepoint of a fifteen-day batch arrested anaerobic digestion experiment at pH 5.

The measured composition of the nationally representative synthetic food waste mixture was within the range of previously reported values for real food wastes in terms of solids, carbohydrates, proteins, and C:N ratio (Figure 2; Table 3). Large ranges of values are reported for food waste parameters, highlighting the need for standardized feedstock in laboratory studies. The measured composition of the food waste mixture dry mass was 45.38% carbohydrates, 32.51% lipids, and 13.36% proteins, with 333.0 ± 56.0 g COD/kg wet mass, or 346.0 ± 58.2 g COD/L, calculated using the food waste mixture bulk density (Figure 1; Table 3). Inorganic material was 2.76% of dry mass, providing a total mass closure for the measured compositional analysis of 95.83%. Theoretical carbohydrate, lipid, and protein values calculated from USDA FoodData Central data were 50.8%, 29.1%, and 16.9%, respectively, and the theoretical COD was 356 g/kg wet mass, or 370 g COD/L. Thus, measured values were similar to theoretical values.

The measured carbohydrate content of the representative food waste mixture was similar to average values reported in review articles (Figure 1). Carbohydrates were further delineated into more specific categories in the representative mixture. Structural sugars, defined as sugars in extractives-free material, were 45.34% of total carbohydrates, with non-structural sugars as 0.04%. Whole starches and lignin were 29.46% and 3.32% of dry mass, respectively, which fall within the ranges of 22.7 ± 13.7% starches and 5.8 ± 5.1% lignin reported in the review by Moonsamy et al. [16].

The measured and theoretical lipids values for the synthetic food waste were greater than what has often been reported for food wastes, with the addition of waste fats and oils as the likely driver of this difference. The theoretical lipids content of the mixture without the added vegetable oil is 15.6% of the dry mass, which aligns well with previously reported values shown in Figure 1. Poe et al. [12] observed that lipid contents of university dining hall waste varied from 0.1 to 37.9% over a 16-week period, indicating that the lipids content of 32.51% is higher than average but not outside the range of reported values of real wastes. However, wasted oils and fats are often collected separately at restaurant, food service, and household levels as yellow grease or brown grease. These lipids should be included as food losses at the retail and consumer levels and were included here in the food waste mixture design.

However, yellow and brown grease are not always captured with food waste collected and characterized in the literature. While greases do have other applications for biofuels production [26], they are suitable feedstocks for AD and AAD [27]. To be representative of a scenario where greases are captured and used separately from food waste, Table A1 includes a recipe without the contribution of oil to total food losses. The theoretical composition of the mixture without oil addition is 61.7% carbohydrates, 20.4% proteins, and 15.1% lipids.

The food waste mixture was prepared with ingredients that were purchased as both raw (e.g., meats) and cooked (e.g., canned vegetables). Cooking food, or applying thermal pretreatment, can improve the hydrolysis of lignocellulosic material in food waste and improve methane yield in AD studies [28]. In reality, a portion of wasted food would be cooked; however, there is a lack of high-quality data that splits food waste into cooked vs. raw across the food supply chain. Further studies should explore the impact of cooking foods on AD and AAD performance, as cooking food changes the physiochemical composition [29]; however, obtaining data representative of performance at scale would be predicated on first obtaining data on the fraction of food waste that is cooked vs. raw for each food waste category. Inability to consider the physicochemical state of the foods by category is a limitation of the current method that should be considered.

**Table 3:** Comparison of synthetic food waste with previously reported food waste.

Food Waste Source	City, Country	Volatile Solids (g/L or %)	C:N Ratio	BMP (mL CH_4_/g VS)	Reference
Nationally representative synthetic mixture	United States	24.2 ± 0.1% (97% of TS)	17.1	495 ± 44 (average ± stdev)	Present study
Review of reported literature values	Several	90.93 ± 5.38% of TS	19.7 ± 9.2	–	[16]
Review of literature reported values	Several	92.5 ± 5.3% of TS	–	220–530	[31]
University dining hall, composite food waste	Leeds, United Kingdom	92.9% of TS	10.95	393–543	[30]
University dining hall	Cork, Ireland	95.3% of TS	14.2	433–539	[11]
Review of collected food wastes	Several	90.9 ± 3.6% of TS	18.4 ± 3.4	260–529	[32]
Measurement of as collected food wastes mixed with soiled paper	Florida, Georgia and North Carolina, USA	–	–	328 ± 80, max of 538	[33]

The COD of the nationally representative synthetic mixture was higher than COD values reported for many real world food wastes [16], but was similar to university dining hall waste collected by [30]. The higher lipid content of the synthetic food waste mixture likely contributed to the higher COD, since lipids have the highest COD per gram of dry weight among the macronutrients. Additionally, the relatively high COD likely reflects the use of fresh food wastes, in which readily degradable organic matter had not been reduced by microbial activity prior to collection.

Measured protein content was lower than the calculated theoretical value but was within a standard deviation of several average values reported in the literature (Figure 1). Higher theoretical protein content is most likely due to a greater nitrogen-to-protein conversion factor of 6.25 typically used in the USDA FoodData Central database as compared to the 4.6 nitrogen-to-protein conversion factor used to calculate the measured value herein. Nitrogen-to-protein conversion factors vary by foodstuff, with 6.25 often being an overestimate [34]. Recalculating the theoretical protein content using a nitrogen-to-protein conversion factor of 4.6 instead of 6.25 changed the value from 16.9% to 12.4%, more closely aligning with the measured value of 13.36%. The measured nitrogen content of 2.9% and C:N ratio of 17.1 agree with other reported values [32], showing agreement that the conversion factor used for proteins is likely driving the differences between theoretical and measured protein values.

### 3.2. Bench Scale Digestion Studies

#### 3.2.1. Biochemical Methane Potential Study

The BMP yield for the food waste mixture was 495 ± 44 mL CH_4_/g VS, which falls within the ranges reported for food wastes in the literature (Table 3). This result indicates that the synthetic food waste mixture is representative of real food waste in terms of total digestible energy content. Notably, BMP values can vary based on use of acclimated inoculum sources [11] and inoculum-to-substrate ratios [30]. Here, the mixture was finely blended to reduce particle size, prepared from fresh foods that had not undergone degradative losses during storage, and exhibited a higher COD than many reported food wastes; therefore, the BMP was expected to fall toward the upper end of reported values. Reduction in particle size has been shown to improve the BMP yield of food wastes [30,35]. We reduced particle size to achieve homogeneity for small-scale batch studies, but the food waste can be maintained in larger pieces for application in larger scale reactors.

#### 3.2.2. Arrested Anaerobic Digestion Batch Study

Similar total carboxylic acid (TCA) titers and yields were observed when the food waste mixture was digested alone as compared to food waste mixture co-digested with AD sludge as an inoculum source, but the two groups produced different product profiles (Figure 2). An average TCA titer of 19.7 ± 1.8 g/L was reached in the food waste + AD sludge, compared to 18.1 ± 3.8 g/L TCA reached in the food waste only group. Average TCA yields, per gram of total VS fed per group, were 0.83 ± 0.10 g/g VS in food waste + AD sludge and 0.94 ± 0.21 g/g VS in the food waste only group.

While the food waste mixture was able to act as its own source of inoculum, a more advantageous product profile containing more short- and medium-chain fatty acids, including heptanoic and caproic acids, was obtained when an external inoculum source was used. The total fatty acid (TFA) titer reached in the food waste + AD sludge group was 18.8 ± 2.4 g/L, which was greater than the 9.6 ± 3.8 g/L TFA produced by the food waste only reactors. Inoculum source has been shown to affect product profile in AAD [36], and [37] also found that the co-digestion of food waste with wastewater sludge increased the production of caproic acid. The optimization of reactor operations and conditions is a key area of study for AAD improvement, and the values reported herein are an example dataset to which improvements can be compared. Due to the reproducibility of the synthetic food waste mixture as a feedstock, changes in product profile and titer can be disentangled from feedstock variation and accurately attributed to process changes. Subsequent studies with real food waste will be necessary to validate the applicability of results obtained with synthetic formulations under more realistic conditions.

### 3.3. Expansion of Approach to Other Regions

This synthetic food waste mixture was based on United States food loss data and may not be representative of food waste in other countries. A similar approach could be taken for other countries, or even regions such as provinces or states. The core approach of this food waste mixture design was using defensible and publicly available food loss data to create a representative and reproducible food waste mixture. This methodology can be used for any country or region for which food loss data are available. Additionally, the basis for the mixture characterized herein, the LAFA database, is updated over time; more recent data will become available in the future, and the mixture could be updated accordingly. Future work could include expanding this approach to other countries, or regions within one country, and comparing values and performance between standardized mixtures.

## Figures and Tables

**Table 1:** Composition of and foods selected for United States nationally representative synthetic food waste using USDA LAFA data.

Food Group	Total Retail and Consumer Level Losses (%)	Representative Food Selected	Contribution to Food Waste Mixture (%)
**Grain products**	13.9	Quick 1 min oatmeal	6.95
		Toasted oat cereal	6.95
**Fruit**	13.9		
Fresh	10.4	Granny smith apples	5.2
	10.4	Bananas	5.2
Processed	3.4	Canned peaches	3.4
**Vegetables**	18.9		
Fresh	13.5	Green leaf lettuce	6.75
		Raw carrots	6.75
Processed	5.3	Canned green beans	2.65
		Canned diced tomatoes	2.65
**Dairy products**	19.2		
Liquid milk	12.8	Whole fat milk	12.8
Other dairy products	6.4	Fat-free yogurt	3.2
		Cheddar cheese	3.2
**Meat, poultry, and fish**	11.6		
Meat	6.5	Ground beef (80% lean, 20% fat)	3.25
		Boneless porkchops	3.25
Poultry	3.6	Chicken thigh (boneless and skinless)	3.6
Fish and seafood	1.4	Tilapia	1.4
**Eggs**	2.1	Eggs	2.1
**Tree nuts and peanuts**	0.4	Roasted unsalted peanuts	0.2
		Sliced almonds	0.2
**Added sugar and sweeteners**	12.6	Sweet bread: assorted	12.6
** Added fats and oils**	7.4	Canola oil	7.4

**Table 2:** Batch arrested anaerobic digestion experimental design.

Group	Total Food Waste Volume Fed (mL)	AD Sludge Volume (mL)	Deionized Water Volume (mL)
Food waste only	75	—	225
Food waste + AD sludge	75	225	—
AD sludge control	—	225	25

## Data Availability

Data available upon request.

## Abbreviations

The following abbreviations are used in this manuscript:

AD	Anaerobic digestion
AAD	Arrested anaerobic digestion
BMP	Biomethane potential
COD	Chemical oxygen demand
sCOD	Soluble chemical oxygen demand
C:N	Carbon to nitrogen ratio
LAFA	Loss-adjusted food availability database
TCA	Total carboxylic acid
USDA	United States department of agriculture
VS	Volatile solids
WRF	Water reclamation facility

## Appendix A

**Table A1:** Nationally representative food waste mixture for the United States based on LAFA data, without added oil.

Food Group	Total Retail and Consumer Level Losses (%)	Representative Food Selected	Contribution to Food Waste Mixture (%)
**Grain products**	15.0	Quick 1 min oatmeal	7.5
		Toasted oat cereal	7.5
**Fruit**	15.0		
Fresh	11.3	Granny smith apples	5.65
		Bananas	5.65
Processed	3.7	Canned peaches	3.7
**Vegetables**	20.5		
Fresh	14.6	Green leaf lettuce	7.3
		Raw carrots	7.3
Processed	5.8	Canned green beans	2.9
		Canned diced tomatoes	2.9
**Dairy products**	20.7		
Liquid milk	13.8	Whole fat milk	13.8
Other dairy products	6.9	Fat-free yogurt	3.45
		Cheddar cheese	3.45
**Meat, poultry, and fish**	12.5		
Meat	7.0	Ground beef (80% lean, 20% fat)	3.5
		Boneless porkchops	3.5
Poultry	3.9	Chicken thigh (boneless and skinless)	3.9
Fish and seafood	1.5	Tilapia	1.5
**Eggs**	2.3	Eggs	2.3
**Tree nuts and peanuts**	0.4	Roasted unsalted peanuts	0.2
		Sliced almonds	0.2
**Added sugar and sweeteners**	13.6	Sweet bread: assorted	13.6
